# Isolation and Characterization of a Novel Sialoglycopeptide Promoting Osteogenesis from *Gadus morhua* Eggs

**DOI:** 10.3390/molecules25010156

**Published:** 2019-12-30

**Authors:** Zhiliang Hei, Meihui Zhao, Yingying Tian, Hong Chang, Xuanri Shen, Guanghua Xia, Jingfeng Wang

**Affiliations:** 1 Hainan Engineering Research Center of Aquatic Resources Efficient Utilization in South China Sea, Hainan University, Hainan 570228, China; 2 College of Food Science and Technology, Hainan University, Hainan 570228, China; 3 Marine Biomedical Research Institute of Qingdao, Qingdao 266003, China; 4 Hainan Institute for Food Control, Hainan 570228, China; 5 College of Food Science and Engineering, Ocean University of China, Qingdao 266003, China

**Keywords:** *Gadus morhua* eggs, sialoglycopeptide, osteogenesis, structure characterization

## Abstract

*Gadus morhua* eggs contain several nutrients, including polyunsaturated fatty acids, lecithin and glycoproteins. A novel sialoglycopeptide from the eggs of *G. morhua* (*Gm*-SGPP) was extracted with 90% phenol and purified by Q Sepharose Fast Flow (QFF) ion exchange chromatography, followed by S-300 gel filtration chromatography. *Gm*-SGPP contained 63.7% carbohydrate, 16.2% protein and 18.6% N-acetylneuraminic acid. High-performance size exclusion chromatography and sodium dodecyl sulfate-polyacrylamide gel electrophoresis (SDS-PAGE) demonstrated that *Gm*-SGPP is a 7000-Da pure sialoglycopeptide. β-elimination reaction suggested that *Gm*-SGPP contained N-glycan units. Amino acid N-terminal sequence analysis indicated the presence of Ala-Ser-Asn-Gly-Thr-Gln-Ala-Pro amino acid sequence. Moreover, N-glycan was connected at the third Asn location of the peptide chain through GlcNAc. *Gm*-SGPP was composed of D-mannose, D-glucuronic acid and D-galactose. Fourier transform-infrared spectroscopy (FT-IR), ^1^H-nuclear magnetic resonance spectroscopy (^1^H-NMR) and methylation analysis were performed to reveal the structure profile of *Gm*-SGPP. In vitro results showed that the proliferation activity of MC3T3-E1 cells was significantly promoted by *Gm*-SGPP. In vivo data revealed that *Gm*-SGPP increased the calcium and phosphorus content of tibias and promoted longitudinal bone growth in adolescent rats.

## 1. Introduction

Gadidae *Gadus morhua* is one of the most commercially important fish species, with an annual marine catch of 8 million tons in China. Several by-products are generated during fish processing, most of which are used in the production of low-value products. Fish eggs are rich in all nutrients required for embryo development and many biologically active substances, including docosahexaenoic acid, eicosapentaenoic acid, lecithin and glycoproteins [1,2]. Furthermore, fish eggs have been reported to be able to improve memory [3], alleviate metabolic syndrome [4] and inhibit osteoclastogenesis [5]. However, high-value utilization of fish eggs is yet to be fully developed.

Glycoproteins are polysaccharide–peptide or polysaccharide–protein complexes of varying structures and bioactivities when compared to those of polysaccharides [6,7,8,9]. The analysis of glycopeptide structure involves polysaccharide chain elucidation prior to that of the peptide chain [10] as well as monosaccharide composition, glycosidic linkage configuration, monosaccharide sequence [11] and secondary and 3D structure determination [12]. Sialic acid, a nine-carbon monosaccharide derivative, is often located at the terminal monosaccharide of glycoproteins existing in various biological tissues [13,14] and forms an important basis of the glycoprotein/glycopeptide functional diversity [15,16].

Fish eggs contain glycoproteins/glycopeptides, some of which are known to have a particularly high sialic acid content. Following purification of the first sialoglycoprotein from rainbow trout [17], several studies demonstrated that sialoglycoproteins also exist in various fish species, such as Medaka fish, salmon and Pacific herring [17,18,19,20]. Further, the structure of sialoglycoprotein in the various fish species is different. Seko [21] isolated a 100–120 kDa sialoglycoprotein and a 6 kDa sialoglycopeptide from flounder eggs. To date, most studies have focused on the glycoprotein structure but its bioactivity has been largely ignored. Recently, we found that sialoglycoproteins from freshwater *Carassius auratus* eggs had anti-osteoporotic effects through the promotion of osteogenesis and the inhibition of bone resorption [22]. Kang [23] reported that egg yolk protein promotes longitudinal bone growth in adolescent rats. Herein, we elucidated the structure and biological activity of glycopeptides in *G. morhua* eggs.

In the present study, a novel glycopeptide from the eggs of *G. morhua* (*Gm*-SGPP) was isolated and purified and its structure profile was identified through a combination of spectroscopic techniques and amino acid sequencing methods. Furthermore, in vitro cell proliferation and in vivo bone growth promotion abilities of the glycopeptide were investigated.

## 2. Results and Discussion

### 2.1. Characterization of Gm-SGPP

**Figure 1:**
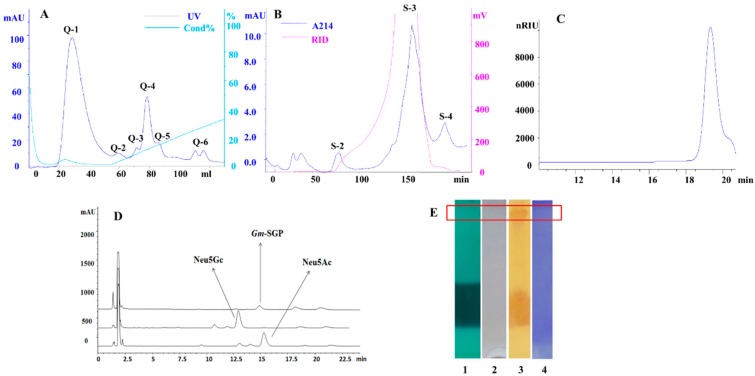
Chromatography isolation, purification and characterization of *Gm*-SGPP. (**A**) Anion-exchange chromatography of crude sample on Q Sepharose Fast Flow column (QFF) (3 × 5 mL). Flow rate, 1 mL/min; fraction size, 4 mL. (**B**) Gel filtration of fraction Q-4 on Sephacryl S-300 column (1.6 × 100 cm). Flow rate, 1 mL/min; fraction size, 4 mL. (**C**) High-performance size exclusion chromatography (HPSEC) profiles of *Gm*-SGPP using a TSK-GEL G4000PWXL column (30 cm × 7.8 mm). Flowing phase, 0.2 M NaCl; flow rate, 0.5 mL/min; column temperature, 40 °C (**D**) High-performance liquid chromatograph (HPLC) of sialic acid using a ZORBAX SB-C18 column (4.6 mm × 150 mm). Flowing phase, 5% acetonitrile–ultrapure water; flow rate, 1 mL/min; column temperature, 35 °C (**E**) Sodium dodecyl sulfate-polyacrylamide gel electrophoresis (SDS-PAGE)of *Gm*-SGPP. 1, green for phosphorus staining; 2, Sudan black B for lipid staining; 3, periodic acid/Schiff for carbohydrate staining; 4, Coomassie Brilliant Blue for protein staining.

*Gm*-SGPP was initially characterized using sodium dodecyl sulfate (SDS)-polyacrylamide gel electrophoresis (PAGE) and high-performance size exclusion chromatography (HPSEC). The single peak observed in the HPLC spectrum indicated that *Gm*-SGPP was a pure glycopeptide with an estimated molecular weight of 7000 Da (Figure 1C). It is, therefore, a much smaller glycoprotein compared to that isolated from rainbow trout, which has a molecular weight of 260 kDa. In SDS-PAGE, *Gm*-SGPP stained positively for carbohydrate but weakly for lipid and phosphorus (Figure 1E), indicating that *Gm*-SGPP is a pure and single glycopeptide. High-performance liquid chromatography (HPLC) results showed that the appearance time of *Gm*-SGPP was consistent with that of Neu5Ac at 19.32 min but not for Neu5Gc at 13.12 min (Figure 1D). This indicated that Gm-SGPP contained Neu5Ac. Kitajima [20] found that glycoproteins in *Salmo gairdneri* contained high amounts of Neu5Gc. Seko [21] reported that sialic acids are not present in the glycoprotein of *Paralichthys olivaceus*, indicating that the type of sialic acid is different in glycoprotein/peptide from different species.

### 2.2. Chemical Composition

**Figure 2:**
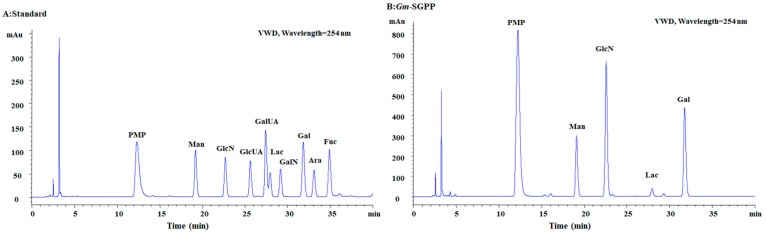
HPLC chromatograph of *Gm*-SGPP monosaccharide composition. (**A**) Seven neutral monosaccharides as control. (**B**) *Gm*-SGPP.

The main feature of *Gm*-SGPP was found to be Neu5Ac acid, accounting for 18.6% of its weight. Additionally, *Gm*-SGPP was composed of 16.2% protein and 63.7% hexose. As shown in Figure 2, monosaccharide composition analysis showed that *Gm*-SGPP was comprised of Man, GlcN and Gal. Small but distinct differences in carbohydrate composition are found in the glycoprotein from the yolk in hen eggs [24].

### 2.3. Determination of Glycosidic Bond Type

**Figure 3:**
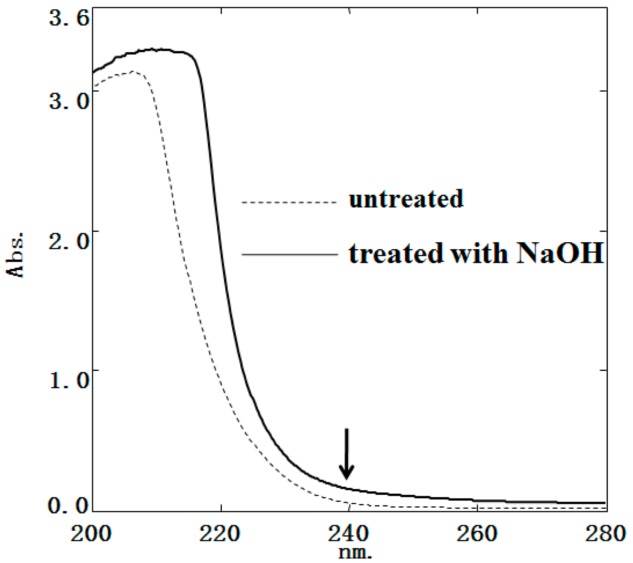
UV spectrum of *Gm*-SGPP treated and untreated with NaOH. Wavelength, 200–400 cm^−1^.

N- and O-glycosylation are the two main types of protein glycosylation. Herein, the absorption of *Gm*-SGPP, recorded at 240 nm, treated with NaOH remained unchanged (Figure 3), suggesting the absence of O-glycosylation. Combining the results of amino acid composition and N-terminal amino acid sequence analysis, *Gm*-SGPP was deduced to be a glycoprotein with N-glycosylation. Iwasaki [25] reported that the glycophosphoproteins of Medaka fish possess N-linked glycan units. Seko [21] reported that the glycosidic bond in *P. olivaceus* was N-linked glycan and did not contain sialic acid in the carbohydrate chain. These results indicated that glycosidic bond and carbohydrate chain are different among species.

### 2.4. Amino Acid Composition and Sequence Analysis of Gm-SGPP

*Gm*-SGPP was observed to be composed of seven amino acids (Table 1). High amounts of aspartate further indicated that *Gm*-SGPP contained N-glycan units.

*Gm*-SGPP was subjected to automated amino acid sequence analysis, revealing the sequence to be NH_2_-Ala-Ser-X-Gly-Thr-Gln-Ala-Pro. The third cycle revealed no phenylthiohydantoin derivatives, indicating that Asn contained an N-linked glycan chain. It was, thus, concluded that the peptide portion of *Gm*-SGPP consisted of NH_2_-Ala-Ser-Asn(CHO)-Gly-Thr-Gln-Ala-Pro. This result is similar to that of the sialoglycoproteins isolated from Medaka eggs [26], wherein the fifth position of Asn was attached with the glycan moiety.

### 2.5. Fourier Transform-Infrared (FT-IR) Spectroscopy Analysis of Gm-SGPP

**Figure 4:**
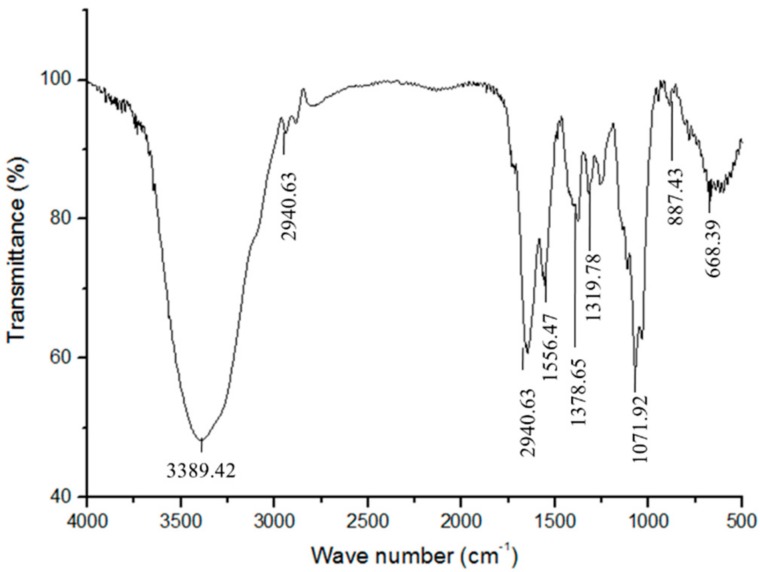
IR spectrum of *Gm*-SGPP. *Gm*-SGPP was mixed with KBr powder and measured in the range 4000–400 cm^−1^.

Fourier transform infrared (FT-IR) spectra showed a strong and broad absorption peak at 3500–3200 cm^−1^ corresponding to the N–H stretching vibrations of proteins and hydroxyl stretching vibration of oligosaccharide (Figure 4) [27]. The bands at 2940.63 and 1114.48 cm^−1^ were attributed to C–H and C–O stretching vibrations, respectively. The wave numbers between 1200 and 800 cm^−1^ represented the finger print region for carbohydrates. The peak at 1071.92 cm^−1^ was assigned to the CH_2_–O–CH_2_ stretching vibration. All the above bands are characteristics of oligosaccharides. The peaks at 2940.63 and 1556.47 cm^−1^ are the characteristic absorption bands for proteins, attributed to C=O stretching and N–H bending vibrations of the acylamino group, respectively [6,28]. These data consequently further demonstrated that *Gm*-SGPP is a glycopeptide.

### 2.6. Methylation Analysis of Gm-SGPP

Methylation analysis of the *Gm*-SGPP glycan portion was performed to investigate the inter-glycosidic linkages between monosaccharide residues [29,30]. The partially methylated alditol acetate derivatives (PMAAs) of *Gm*-SGPP were analyzed by gas chromatography (GC)–mass spectrometry (MS). The results were classified according to the database of the University of Georgia Complex Carbohydrate Research Center. GC–MS analysis results are shown in Table 2. At 36.37 min, the characteristic fragment ions of 1,2,4,5-Ac4-2,3,6-Me_3_-GlcNAc were detected, demonstrating that *Gm*-SGPP contained →4) GlcNAc (1→. Furthermore, it was found that →2,4)Man(1→ and →3,6)Man(1→were present as the fragment ions of 1,2,4,5-Ac_4_-3,6-Me_2_-Man and 1,5,6-Ac_3_-2,4-Me_2_-Man, respectively. At 31.31, 22.43, 24.80 and 23.59 min, the markers of →3,4) Gal (1→ Gal (1→, →3)Gal(1→ and →4)Gal(1→ were observed. In conclusion, *Gm*-SGPP was in accordance with the specific regular “five core carbohydrate.” Taguchi [25] found that →4)GlcNAc(1→, →2,4)Man(1→, →3,6)Man(1→ and →3)Gal(1→ are present in *Oryzias melastigma* and Neu5Ac is attached at the end of Gal.

### 2.7. H-Nuclear Magnetic Resonance (^1^H-NMR) Spectroscopy of Gm-SGPP

**Figure 5:**
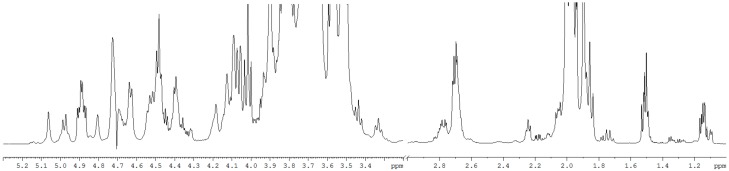
^1^H-nuclear magnetic resonance spectroscopy (^1^H-NMR) spectrum of *Gm*-SGPP. *Gm*-SGPP was dissolved in 1 mL of D_2_O and spectra were obtained at 600 MHz with sufficient acquisition time.

^1^H-nuclear magnetic resonance spectroscopy (^1^H-NMR) (^1^H-NMR) spectrum of *Gm*-SGPP is shown in Figure 5. According to Seko [24], ^1^H-NMR signals for H-3ax and H-3eq of Neu5AC appear at 1.713–1.721 ppm and 2.666–2.672 ppm, respectively. In our experiments, the signals for Neu5AC were located at 1.717 and 2.671 ppm, suggesting that the Neu5AC residues of *Gm*-SGPP have an α-(2–6) linkage to galactose. Further, the signal at 5.81 ppm was assigned to the GlcNAcβ(1)-Asn residue. The anomeric hydrogen signals between 4.61 and 4.65 ppm were attributed to GlcNAc-2,5,5′,5′′,5′′ of H1 and the signal at 4.86 ppm was attributed to GlcNAc-1-H1. The signals of Man-3-H1, Man-4-H1 and Man-3-H2 appeared at 4.77, 5.07 and 4.18 ppm, respectively. The above data further proved that the carbohydrate chain of *Gm*-SGPP is a four-branched chain, which was consistent with methylation analysis results [13].

**Figure 6:**
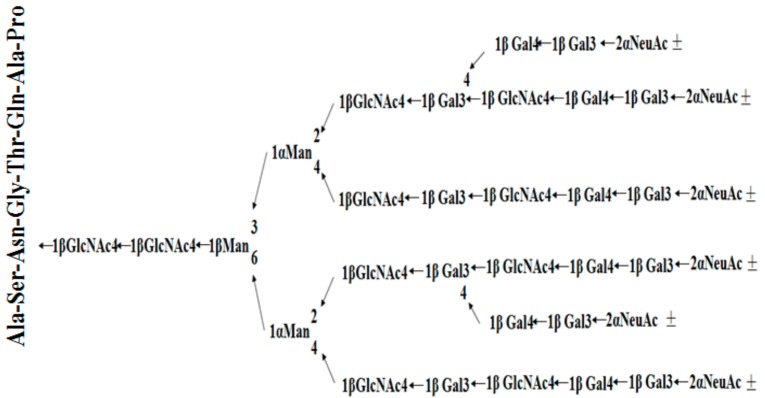
Structure outline of *Gm*-SGPP.

In summary, combining the results of the peptide and glycan moieties, we speculated that the structure outline of *Gm*-SGPP is as shown in Figure 6.

### 2.8. Gm-SGPP Promoted MC3T3-E1 Cell Proliferation

**Figure 7:**
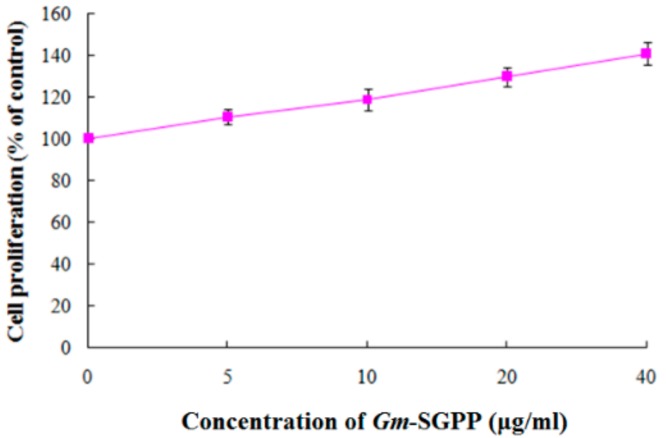
Effects of *Gm*-SGPP on growth of MC3T3-E1 cells. MC3T3-E1 cells were treated with various concentrations of *Gm*-SGPP (0, 5, 10, 20 and 40 µg/mL) for 72 h. *Gm*-SGPP promoted the proliferation of MC3T3-E1 cells in a dose-dependent manner.

MC3T3-E1 is a well-recognized pre-osteoblastic cell line with biological characteristics, including alkaline phosphatase and type I collagen activity, of osteoblasts [31]. MC3T3-E1 is often used as a cell model of bone metabolism research. Kim [32] reported that water-soluble yolk protein from chicken eggs promotes MC3T3-E1 cell proliferation, differentiation and mineralization, thus effectively preventing bone loss. The present results showed that the proliferation activity of MC3T3-E1 cells was significantly promoted by *Gm*-SGPP in a dose-dependent manner (Figure 7), indicating that *Gm*-SGPP may have anti-osteoporotic effects by promoting bone formation. Further studies on the bioavailability of *Gm*-SGPP are required.

### 2.9. Gm-SGPP Promoted Bone Formation in Adolescent Mice

**Figure 8:**
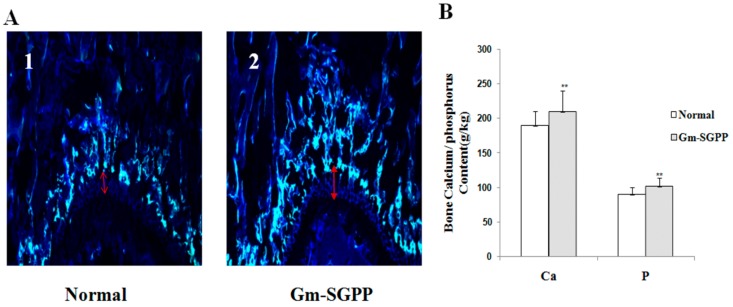
(**A**) Effect of *Gm*-SGPP on longitudinal bone growth in adolescent male mice. Fluorescence photomicrographs of longitudinal sections at the growth plate in the distal femurs. The fluorescent line corresponds to the injection of tetracycline hydrochloride (20 mg/kg body weight), which binds with calcium and can be detected by ultraviolet illumination. The arrow between the fluorescent line formed by tetracycline and the epiphyseal end line of the growth plate indicates the length of bone growth during 48 h (10× magnification). Normal (1) and *Gm*-SGPP (100 mg/kg) groups (2). (**B**) Effect of *Gm*-SGPP on calcium and phosphorus content of tibias in adolescent male mice. Normal and *Gm*-SGPP mice were represented as the normal control group (treated with normal saline) and *Gm*-SGPP-treated group (100 mg/kg body weight), respectively. Mice were treated for 4 weeks and tibias were collected at −20 °C to measure calcium and phosphorus content. Data are expressed as mean ± SD (*n* = 10/group). Multiple comparisons were performed by one-way ANOVA analysis. *p < 0.05, **p < 0.01 versus the control.

Bone growth was assessed during a 48 h period using tetracycline hydrochloride labeling on the newly formed bone to evaluate the rate of bone growth. Longitudinal bone growth increased significantly by *Gm*-SGPP treatment compared with that by the control group (Figure 8A). Changes in bone calcium and phosphorus content directly reflect the density of bone. In this study, compared with the control, bone calcium and phosphorus contents of the *Gm*-SGPP group increased markedly by 10.42% and 13.12%, respectively, in mouse tibia (Figure 8B). In conclusion, *Gm*-SGPP efficiently promoted bone formation in adolescent mice compared to that in the control [23]. In a previous study, Leem et al. found that the yolk water-soluble protein (100 mg/kg) of hen eggs can improve the longitudinal bone growth of adolescent male rats, while milk protein and casein cannot ameliorate the longitudinal bone growth. We speculated that the functional factor of yolk water-soluble protein (100 mg/kg) of hen egg may associated with sialoglycopeptide from hen egg and this needs further study.

## 3. Methods

### 3.1. Materials

Fresh mature female *G. morhua* were purchased from Oriental Ocean Company of China and stored at −80 °C until further use. QFF and Sephacryl S-300 were obtained from GE Healthcare (Fairfield, CT, USA). Neu5Ac, Neu5Gc, D_2_O, 1-phenyl-3-methyl-5-pyrazolone and dextran standards were purchased from Sigma (St. Louis, MO, USA). Dulbecco’s modified Eagle medium (DMEM) was obtained from Gibco (Gaithersburg, MD, USA). Fetal bovine serum was from HyClone (Logan, UT, USA).

### 3.2. Preparation of Gm-SGPP

Unless otherwise stated, all extraction procedures were performed at 4 °C. Fresh eggs were homogenized in a blender with 3 volumes (*v*/*w*) of 0.4 M NaCl for 30 min and then centrifuged at 4500 rpm for 10 min. The supernatant was then dialyzed for 24 h to obtain water-soluble glycopeptides. An equal volume of 90% phenol was added to the supernatant with mild stirring overnight to destroy the protein 3D structure while retaining glycoprotein stability. After centrifugation, the aqueous phase was dialyzed and lyophilized and the crude samples were then applied to a QFF column (GE Healthcare, USA) using the AKTA™ UPC 100 (GE Healthcare) system eluted by a linear gradient (0–1 M) of NaCl in 0.02 M Tris-HCl buffer (pH 8.0). The elution profile was monitored by measuring the absorbance at 230 nm for proteins. The carbohydrate profile was assayed using the phenol–sulfuric acid method. As shown in Figure 1A, the Q-4 fraction, sialic acid-rich fraction, was further chromatographed using a Sephacryl S-300 column (GE Healthcare) and eluted with ultrapure water. The S-3 fraction containing the most protein, carbohydrates and sialic acid was freeze-dried and used as the experimental sample.

### 3.3. Identification of Gm-SGPP

The molecular weight of *Gm*-SGPP was determined by HPSEC. Measurements were performed on a TSK-GEL G4000PWXL column (TOSOH BIOSEP) eluted with 0.2 M NaCl. A calibration curve was obtained with a series of dextran standards. Glycopeptide characteristics and purity were evidenced by SDS-PAGE and HPSEC. *Gm*-SGPP was stained by periodic acid–Schiff for carbohydrates [33,34], Sudan black B for lipids [35] and methyl green for phosphorus [36]. The type of sialic acid was identified by HPLC [37] using Neu5AC and Neu5GC as standards.

### 3.4. Chemical Composition of Gm-SGPP

The carbohydrate content of *Gm*-SGPP was determined using the phenol–sulfuric acid method using glucose as the standard [38]. Monosaccharide composition was analyzed through the PMP precolumn derivatization with HPLC (PMP-HPLC) method using a ZORBAX Eclipse XDB-C18 column (Agilent, USA) [39]. Protein content was measured by the Folin’s reagent method using bovine serum albumin as the standard [40]. Neu5Ac content determination was as described above.

### 3.5. Determination of Gm-SGPP Glycosylation Type

Glycosylation type was determined through the β-elimination reaction [41]. Briefly, *Gm*-SGPP (5 mg) was dissolved in 5 mL of 0.2 M NaOH solution for 2 h, followed by UV-vis spectra determination using a UV-2550 spectrophotometer (Shimadzu, Japan) within the 200–400 cm^−1^ wave range. The sample without NaOH treatment was used as the control.

### 3.6. FT-IR Spectroscopy Analysis of Gm-SGPP

The FT-IR spectrum of *Gm*-SGPP was recorded with a Nicolet Magna-IR 550 spectrometer within the 4000–400 cm^−1^ range using the potassium bromide (KBr) pellet method.

### 3.7. Amino Acid Composition Analysis of Gm-SGPP

The amino acid composition was analyzed using a Hitachi 835-50 model amino acid analyzer (Hitachi, Japan) following hydrolysis in 6 M HCl at 110 °C for 24 h under N_2_.

### 3.8. Amino Acid Sequence Analysis of Gm-SGPP

Deglycosylation of *Gm*-SGPP was performed according to the method of Edge [42]. Briefly, 20 mg of dry *Gm*-SGPP was added to a 2-mL mixture of anisole and trifluoromethanesulfonic acid and nitrogen was bubbled through the solution, followed by magnetic stirring at 0 °C for 30 min. After the reaction, 50-fold of diethyl ether and 95% ethanol were added to obtain protein precipitation. The protein was re-dissolved in distilled water and applied to a Superdex^TM^ Peptide S-100 GL column (GE Healthcare, USA) and eluted with 0.2 M NH_4_HCO_3_ at a flow rate of 0.4 mL/min. Deglycosylated *Gm*-SGPP was collected for amino acid sequence analysis. The N-terminal amino acid sequence of *Gm*-SGPP was analyzed by an automated Edman degradation performed with a Model PPSQ-33A N-terminal sequencer (Shimadzu, Japan).

### 3.9. Methylation Analysis of Gm-SGPP

Methylation analysis of *Gm*-SGPP was performed according to the method of Hakomori [43]. The sample was subjected to a series of derivative reactions, including methylation, hydrolysis, reduction and acetylation, to obtain PMAAs. PMAAs were analyzed by HP6890II GC–MS (Agilent, USA). Complete methylation was confirmed by the disappearance of the hydroxyl band between 3400 and 3600 cm^−1^ and by the enhanced absorption band of CH_3_ at 2900cm^−1^ in the FT-IR spectrum [44].

### 3.10. ^1^H-NMR Spectroscopy of Gm-SGPP

*Gm*-SGPP (20 mg) was co-evaporated with D_2_O (99.9%) by lyophilization three times and dissolved in 1 mL of D_2_O (99.9%) containing 0.1 µL of 4,4-dimethyl-4-silapentane-1-sulphonic acid as an external standard. ^1^H-NMR spectra of *Gm*-SGPP were obtained using a 600-MHz Bruker AVANCE III spectroscope (Rheinstetten, Karlsruhe, Germany) at 20 °C.

### 3.11. MTT Cell Viability Assay

Pre-osteoblastic MC3T3-E1 cells were obtained from the American Type Culture Collection (Manassas, VA, USA) and cultured in 5% CO_2_ at 37 °C with DMEM supplemented with 10% (*v*/*v*) heat-inactivated fetal bovine serum and 100 g/mL streptomycin. All experiments were repeated at least three times to ensure accuracy.

The proliferation activity of *Gm*-SGPP was determined as follows. MC3T3-E1 cells (3 × 10^3^/well) in the exponential growth phase were seeded in 96-well plates. After 24 h, cells were treated with various concentrations of *Gm*-SGPP (0, 5, 10, 20 and 40 µg/mL) for 72 h. MTT solution (0.5 mg/mL in GI-1640 medium) was added and the cells were incubated for further 4 h. Cell viability was determined by the MTT method.

### 3.12. Animal Treatments

Three-week-old male ICR mice (18.0 ± 2.0 g) were purchased from Vital River Laboratory Animal Center (Beijing, China; Licensed ID: SCXK2012-0001). Three to four mice were housed per cage at 23 ± 1 °C with a 12:12 h light:dark cycle and were provided *ad libitum* access to a standard sterile diet and water. The body weight of these animals was recorded every three days. Animal experiments were approved by the ethical committee of experimental animal care at Ocean University of China.

ICR mice were divided into two groups (*n* = 16 per group)—normal group (treated with physiological saline) and the *Gm*-SGPP-treated group (100 mg/kg body weight of *Gm*-SGPP). Animals in each group were intragastrically administered physiological saline or *Gm*-SGPP (1 mL/100 g body weight) once per day. The mice were sacrificed after four weeks of treatment to obtain the femurs and tibias used in subsequent experiments.

Bone growth rate was measured by measuring the gap between the fluorescent line formed by tetracycline hydrochloride and the epiphyseal end line of the femur growth plate using a fluorescence microscope (Leica DM2500, Leica). Tetracycline hydrochloride (20 mg/kg body weight) was subcutaneously injected to mice 48 h prior to sacrifice. The dissected femurs were fixed in 10% neutral formaldehyde for 24 h, decalcified by immersion in 10% Ethylene diamine tetraacetic acid(EDTA) solution for 3 h and sectioned at a thickness of 10 μm. The calcium and phosphorus contents of tibia were measured by inductively coupled plasma-optical emission spectrometry (ICP-OES; 7300DV, PerkinElmer). The dissected tibias were crushed and dried at 105 ± 1 °C for 2 h, followed by digestion with nitric acid and perchloric acid.

### 3.13. Statistical Analysis

All data were presented as mean ± standard deviation of at least three independent experiments. Statistical comparisons were assessed by one-way analysis of variance (ANOVA), followed by the least significant difference test. All computations were performed using statistical software. Statistical differences were considered significant at *p* < 0.05.

## 4. Conclusions

A sialoglycopeptide from the eggs of *G. morhua* was purified and its structure was elucidated by HPLC, FT-IR, ^1^H-NMR, GC–MS and N-terminal amino acid sequence analysis. It was found that *Gm*-SGPP stimulated the proliferation of pre-osteoblasts and promoted bone formation in adolescent mice. Nevertheless, the biological function of *Gm*-SGPP remains a challenge for future research. *Gm*-SGPP may be an effective supplement in the promotion of bone formation. The present findings will serve as a reference for future study of the structure–activity relationship analysis of *Gm*-SGPP.

## Figures and Tables

**Table 1:** Amino acid composition of *Gm*-SGPP.

Amino Acid	*Gm*-SGPP (mg/g glycopeptide)	Amino Acid	*Gm*-SGPP (mg/g glycopeptide)
Asp	15.33	Gly	16.42
Thr	16.29	Ala	33.51
Ser	15.80	Pro	14.37
Glu	13.90		
Total		125.62	

**Table 2:** Gas chromatography–mass spectrometry (GC–MS) analysis of methylate from *Gm*-SGPP.

Retention Time	Monosaccharide of Methylation	Characteristic Fragment Ions	Connection Type
27.11	1,2,4,5-Ac_4_-3,6-Me_2_-Man	113,130,131,190,233	→2,4)Man(1→
28.23	1,5,6-Ac_3_-2,4-Me_2_-Man	57,87,118,129,189	→3,6)Man(1→
31.31	1,3,4,5-Ac_4_-2,6-Me_2_-Gal	130,190,201,261	→3,4)Gal(1→
22.43	1,5-Ac_2_-2,3,4,6-Me_4_-Gal	57,118,145,161,205	Gal(1→
24.80	1,3,5-Ac_3_-2,4,6-Me_3_-Gal	87,101,118,129,161	→3)Gal(1→
23.59	1,4,5-Ac_3_-2,3,6-Me_3_-Gal	71,118,129,145,161	→4)Gal(1→
36.71	1,2,4,5-Ac_4_-2,3,6-Me_3_-GlcNAc	117,159,233	→4)GlcNAc(1→
